# Evaluation of Physical Activity, Sedentary Patterns, and Lifestyle Behavior in Spanish Preschool Children from the CORALS Cohort

**DOI:** 10.1186/s40798-025-00865-2

**Published:** 2025-06-09

**Authors:** Jose Manuel Jurado-Castro, Belén Pastor-Villaescusa, Cristina Castro-Collado, Mercedes Gil-Campos, Rosaura Leis, Nancy Babio, Luis A. Moreno, Santiago Navas-Carretero, Olga Portolés, Ana Moreira Echeverría, Maria Jose De La Torre-Aguilar, Rosaura Picáns-Leis, Jordi Salas-Salvadó, Pilar de Miguel-Etayo, Katherine Flores-Rojas, Rocío Vázquez-Cobela, Júlia Valero Sales, Maria L. Miguel-Berges, Isabel Izquierdo-López, Carlos Gómez-Martínez, Diana Paola Córdoba-Rodríguez, Gisela Mimbrero, Francisco Jesus Llorente-Cantarero, on behalf of the CORAL Study investigators, Jose Manuel Jurado-Castro, Cristina Castro-Collado, Mercedes Gil-Campos, Rosaura Leis, Nancy Babio, Santiago Navas-Carretero, Maria Jose De La Torre-Aguilar, Rosaura Picáns-Leis, Jordi Salas-Salvadó, Pilar de Miguel-Etayo, Katherine Flores-Rojas, Rocío Vázquez-Cobela, Júlia Valero Sales, Maria L. Miguel-Berges, Isabel Izquierdo-López, Carlos Gómez-Martínez, Gisela Mimbrero, Francisco Jesus Llorente-Cantarero, Helmut Schröder, Ana Moreira, Montse Fitó, Karla A. Pérez-Vega, Mayela Solis Baltodano, Daniel Muñoz-Aguayo, Gemma Blanchart, Sònia Gaixas, María Dolores Zomeño, Isaury Lorenzo, Belén Pastor Villaescusa, Inmaculada Velasco Aguayo, José Luis Castillo Panadero, Rafael Blanco Perea, Susana De la Guerra, Teresa Espino Bermell, Francisca Portero Sánchez, J. Alfredo Martínez, Maria Jesús Moreno-Aliaga, Begoña de Cuevillas García, María Goñi, María Hernández, Salomé Pérez Diez, Carmen Cristobo, Joaquín Escribano, Albert Feliu, Ricardo Closas, Verónica Luque, Natalia Ferré, Irina Gheorghita, Mireia Alcázar, Francisco Martín, Cristina Rey, Ana Pedraza, Olga Salvadó, Marta Ruiz Velasco, José Ángel Bilbao Sustacha, Yolanda Herranz Pinilla, Lidia Rios, María Pascual Compte, Tany E. Garcidueñas-Fimbres, Sara de las Heras-Delgado, Olga Simón, Sònia de la Torre, Càrol Tudela, Sara Moroño García, Alicia López-Rubio, Olga Portoles, Pilar Codoñer Franch, Dolores Corella, Vanessa Martín Carbonell, José V. Sorlí, Luís A. Moreno, Alba Ma Santaliestra-Pasias, Pilar Argente-Arizon, Natalia Gimenez-Legarre, Paloma Flores-Barrantes, Gloria Pérez-Gimeno, Miguel Seral-Cortés, Andrea Jimeno Martinez, Ivie Maneschy

**Affiliations:** 1 Consorcio CIBER, M.P. Fisiopatología de la Obesidad y Nutrición (CIBEROBN), Instituto de Salud Carlos III (ISCIII), Madrid, Spain; 2 University of Cordoba. Maimonides Biomedical Research Institute of Cordoba (IMIBIC), Metabolism and Investigation Unit, Reina Sofia University Hospital., Avda Menéndez Pidal Sn, 14004 Córdoba, Spain; 3 Primary Care Interventions to Prevent Maternal and Child Chronic Diseases of Perinatal and Developmental Origin (RICORS), RD21/0012/0008, Instituto de Salud Carlos III, Madrid, Spain; 4 Unit of Pediatric Gastroenterology, Hepatology and Nutrition, Pediatric Service, Santiago University Clinical Hospital, Santiago de Compostela, Spain; 5 Pediatric Nutrition Research Group, Health Research Institute of Santiago de Compostela (IDIS), Unit of Investigation in Nutrition, Growth and Human Development of Galicia-USC, Santiago de Compostela, Spain; 6 Universitat Rovira i Virgili, Departament de Bioquímica i Biotecnologia. Grup de Recerca en Nutrició, Alimentació, Creixement i Salut Mental, Reus, Spain; 7 Institut d’Investigació Sanitària Pere Virgili (IISPV), Reus, Spain; 8 Growth, Exercise, Nutrition and Development (GENUD) Research Group, University of Zaragoza, Zaragoza, Spain; 9 Instituto Agroalimentario de Aragón (IA2), Universidad de Zaragoza and Instituto de Investigación Sanitaria Aragón (IIS Aragón), Zaragoza, Spain; 10 Center for Nutrition Research, University of Navarra, Pamplona, Spain; 11 IdisNA, Navarra Institute for Health Research, Pamplona, Spain; 12 Department of Preventive Medicine and Public Health, University of Valencia, Valencia, Spain; 13 Fundació Hospital Sant Joan de Deu Martorell, Martorell, Spain; 14 Department of Specific Didactics, Faculty of Education, University of Cordoba, Córdoba, Spain; 15 Departamento de Nutrición y Bioquímica, Facultad de Ciencias, Pontificia Universidad Javeriana, Bogotá DC, Colombia; 16 Ciencias De La Actividad Física y El Deporte, Escuela Universitaria de Osuna (Centro Adscrito a la Universidad de Sevilla), Osuna, Spain; 17 Spanish Network in Maternal, Neonatal, Child and Developmental Health Research (RICORS-SAMID, RD24/0013/0007) Instituto de Salud Carlos III, Madrid, Spain; 18 Neonatology Service, RICORS-SAMID-CIBERER, University Clinical Hospital of Santiago de Compostela, Health Research Institute of Santiago de Compostela (IDIS), Santiago de Compostela, Spain

**Keywords:** Child, 24-h movement, Lifestyle, Physical activity, Sedentary behavior

## Abstract

**Background:**

Physical activity (PA) and sedentary lifestyle are recognized as modifiable risk factors for non-communicable diseases. Healthy habits in early childhood tend to persist throughout life. This study aims to evaluate the physical activity and sedentary behavior patterns in a cohort of Spanish preschool children using device-based measures, and compare these patterns by sex. This study analyzed a sample of 643 preschoolers aged 3–6 years with valid accelerometry data from the Childhood Obesity Risk Assessment Longitudinal Study (CORALS) cohort (NCT06317883; May 30, 2024).

**Results:**

67% of preschoolers met the international PA recommendations, with 72.1 min of moderate-vigorous PA (MVPA) per day. Boys exhibited higher levels of MVPA than girls, and MVPA increased with age in both groups. Sedentary time was higher on weekends, and boys spent more time watching TV than girls. Involvement in extracurricular sports was reported by 67.5% of children, with boys showing a greater engagement in outdoor activities.

**Conclusions:**

A relevant proportion of Spanish preschool children meet the international PA recommendations, with variations based on sex, age, and day of the week, reflecting global trends at this stage of life. Future interventions should address sex-specific preferences and age-related changes to enhance the effectiveness of promoting active lifestyles in this population.

**Supplementary Information:**

The online version contains supplementary material available at 10.1186/s40798-025-00865-2.

## Key Points

- 67% of Spanish preschoolers met international physical activity (PA) recommendations, averaging 72.1 min of moderate-vigorous physical activity (MVPA) per day. Boys exhibited higher levels of MVPA than girls, and both increased their MVPA with age.
- Future interventions should consider sex-specific preferences and age-related changes to effectively promote active lifestyles. Maintaining adequate PA levels and improving adherence is necessary, especially through strategies that encourage greater participation of girls in physical activities.

**Supplementary Information:**

The online version contains supplementary material available at 10.1186/s40798-025-00865-2.

## Background

Physical inactivity is an important modifiable risk factor for non-communicable diseases such as obesity [1]. Despite the World Health Organization's efforts to promote a higher adherence to physical activity (PA) practice offering clear guidelines and adaptable interventions [2], insufficient levels of PA have been reported worldwide [3].

PA habits established in childhood contribute positively to the child's development and overall health throughout life [4, 5]. International PA guidelines recommend that children aged 3 and 4 engage in at least 180 min of PA per day, including a minimum of 60 min of moderate to vigorous physical activity (MVPA) [6, 7]. For children aged 5 and older, the guidelines advise at least 60 min of MVPA per day [2]; along with reducing sedentary time to maximize health benefits. In addition, adequate sleep time for their age is advised to achieve these PA recommendations [8], with a target of approximately 10–13 h per day, while limiting screen time to less than one hour per day [2, 6]. Therefore, it is crucial to closely monitor adherence to all these recommendations, with a particular emphasis on PA levels and duration. This monitoring may serve as a basis for establishing achievable goals, with the aim of enhancing the proportion of the population meeting optimal levels of PA and preventing sedentary behavior [9].

Several intercontinental surveillance systems have been established to monitor and assess adequate levels of PA in children and adolescents worldwide [10]. Traditionally, questionnaires were used to evaluate sedentary behavior and estimate different intensities of PA [11]. However, several methods such as accelerometry have been developed to more accurately assess PA in children and adolescents [12]. This represent a valid, reliable, and practical tool for measuring the intensity, duration, and frequency of movement related to both sedentary time and PA in children [13], and preschoolers [14].

In this context, several initiatives have employed standardized accelerometer protocols to harmonize device-based PA measurements across multiple studies involving children [15, 16]. However, few studies have assessed PA in preschool children using accelerometry [10], and the results have been inconsistent [9, 16, 17].

Furthermore, several factors might contribute to achieving the recommended levels of PA in preschool children. These include the development of motor skills, as well as participation in social activities such as extracurricular sports [18], group activities and active free play [19, 20].

Sex differences also play a significant role in PA levels among preschool children. Boys are generally more physically active than girls, engaging more frequently in vigorous play and organized sports [21]. This disparity can be attributed to a variety of social, cultural, and biological factors, including differing parental encouragement and societal expectations for boys and girls. Understanding these sex-based differences is crucial for designing targeted interventions that promote higher levels of PA among all children, ensuring both boys and girls can meet the recommended levels of PA and enjoy the associated health benefits [21].

Given the limited information on PA patterns and sedentary behavior in preschool-aged children worldwide, the present study aimed to assess PA and sedentary behavior using device-based measures, and their relationships with other related factors in a Spanish preschool-children from the Childhood Obesity Risk Assessment Longitudinal Study (CORALS) cohort.

## Methods

### Study Design and Participants

This cross-sectional study was conducted using data from the CORALS, which followed the Strengthening the Reporting of Observational Studies in Epidemiology (STROBE) reporting guideline. The protocol study was registered (NCT06317883; May 30, 2024).

The CORALS is a long-term prospective observational cohort project focused on preschool children, conducted across seven Spanish centers located in different regions. Its aim is to investigate the risk factors contributing to the development of childhood obesity over a 10-year period (https://corals.es/). Participants were recruited through multiple primary health care centers and schools in the participating cities, ensuring a geographically and socio-demographically diverse sample that captured variability across different contexts. A detailed description of the CORALS is published elsewhere [22]. To be enrolled in the study, parents or caregivers were required to sign a consent form, attend an in-person inclusion visit, and complete several questionnaires at home to provide data on leisure time PA and sociodemographics. The exclusion criteria included families with limited Spanish proficiency or an unstable residence. However, the incompleteness of information provided through questionnaires was not considered a basis for exclusion. All measurements and data collection were carried out across the school year (from September to June).

The Ethics Committee of each recruitment center approved the study protocol (references: 051/2019, 4155/2019, 2019/18, 9/19, 09/2019, 19/27, and 2019/131), which was conducted following the standards of the Declaration of Helsinki [23].

### Clinical Examination and Anthropometric Measurements

Sex was assessed by history and physical examination by the pediatricians. Body weight and body fat mass were measured using a precision scale equipped with an octopolar multifrequency bioelectrical impedance (Tanita MC780SMA; Tanita Europe, B.V.). Height was determined using a portable stadiometer (SECA 213, Scale 20–205 cm; SECA). These assessments were conducted with the child wearing light clothing, without shoes, and following standard procedures. Body mass index (BMI) was calculated as weight divided by height^2^ [24], and BMI Z-score was further calculated based on Spanish reference standards [25]. Waist circumference was measured with a measuring tape (SECA 201), midway between the lowest rib and the iliac crest. The anthropometric measurements were taken by trained personnel, who received specific training before the study to standardize the evaluations, and according to the International Society for the Advancement of Kinanthropometry (ISAK) protocol [26].

### Physical activity evaluation and accelerometry data collection

PA was assessed using ActiGraph GT3X + accelerometers (ActiGraph; Pensacola, FL, USA). The monitor measured accelerations in three individual axes (vertical, horizontal, perpendicular) with a dynamic range of ± 6 units of gravity. Data were collected at a frequency of 30 Hz, with accelerometers programmed to continuously record activity (raw data). To facilitate analysis through the ActiLife software version 6.13.3 (ActiGraph Software Department: Pensacola, FL, USA), the data were then exported into 1-s epochs, minimizing information loss and generating a file in AGD format. The accelerometers were placed on the right side of the waist using an elastic band to ensure the children’s comfort. The parents and teachers were provided with instructions on the correct use of the device and were informed about its purpose and methodology. The device was worn during the school year, not at summer holidays. Furthermore, parents, teachers, and children were instructed that the Actigraph should be worn 24 h per day, for seven days. To account for activities that were not captured by the accelerometers, parents were provided with an activity logbook. They were instructed to record the reason (e.g., showering or swimming) and duration (in time slots) of the activities when they removed the accelerometers. 

Scoring data were processed using ActiLife software with a 60-s epochs conversion [27]. PA and sedentary time were analyzed based on a minimum of ten hours of monitoring per day for at least three days, including one weekend day, which was considered acceptable [27]. Additionally, two criteria were applied to exclude low-quality records: (a) all negative counts were replaced with missing data, and (b) periods of 20 min or more of consecutive zero counts were treated as missing data before downstream analysis [28]. Exclusion criteria for this analysis included invalid data availability, non-compliance with the minimum required monitoring hours, or insufficient recording time on valid days during the week or weekend. The ActiLife data scoring program was used to determine daily min spent in sedentary behavior and PA for each epoch-length dataset, following the activity cut-off points accelerometry protocol by Butte et al. [29], where sedentary time was defined as ≤ 819 counts per min (CPM), light PA was defined as 820–3907 CPM, moderate PA was defined as 3908–6111 CPM, and vigorous PA was defined as ≥ 6112 CPM.

To assess adherence to international PA guidelines, we categorized compliance based on age-specific recommendations [2, 6, 7].

### Active Lifestyle, Extracurricular Sports, Screen Time and Sleeping Patterns

A series of self-administered questionnaires were used to collect information on lifestyle behaviors, completed by parents or caregivers at home. These questionnaires were employed to assess various aspects of children's daily routines, including the PA. Given the specific context of the study and the need to reduce respondents’ burden, these ad hoc questionnaires, based on a previously validated instrument [30] were developed to assess screen time and sleep patterns. This tailored approach was chosen to ensure simple, easy-to-understand questions for parents and caregivers, capturing relevant data while enhancing response rates. To verify consistency, an initial exploratory session was performed, achieving three key objectives: (1) ensuring that each item was appropriate for the study's age range, (2) confirming that each question allowed for accurate and adequate assessment, and (3) validating that each indicator effectively measured its intended construct. Moreover, the exploratory analysis for this tool yielded a Cronbach’s alpha of 0.625, indicating an acceptable level of reliability. Unanswered questions were treated as missing data.

#### Active Lifestyle and Extracurricular Sports

To assess the total weekly time (in hours) spent engaging in sports and physical activities related to an active lifestyle, a previously validated questionnaire was used [30]. This instrument collected data on children’s daily commuting habits, time spent in outdoor play involving PA on school days and weekends, and participation in extracurricular sports activities. For instance, parents were asked, "How much time does your child play outside the home on a weekday/weekend?".

An additional ad hoc questionnaire, previously used and published [22], was employed to assess leisure-time PA, including sedentary behaviors, to determine an active lifestyle. This questionnaire enabled comparisons between groups based on specific movement-related behaviors. Children were categorized based on their compliance (Yes/No) with activities such as non-continuous walks, outdoor family activities, and active or sedentary play (i.e.: running vs. sitting, playing with toys, or coloring). Differences in sedentary time, light PA, and MVPA were analyzed between those who met specific behavioral criteria and those who did not. Only data showing statistically significant differences were presented.

#### Screen Time

Additionally, an ad hoc questionnaire was used to measure the screen time (on school days and weekends) and the child's sleep pattern based on the nighttime sleep and the nap duration during weekdays, weekend days, or holidays. Screen time on weekdays and weekend days was assessed by two questions: (a) “How long does your child watch television?” and (b) “How long does your child play in the computer/cell phone/game console?” Possible answers were “none,” “0.5–1 h/day,” “1–2 h/day,” “2–3 h/day,” “3–4 h/day,” or “more than 4 h/day.” Total screen time was calculated as a quantitative variable and assessed continuously (hours/day). Compliance with screen time recommendations was categorized dichotomously based on existing guidelines. For children under 5 years old, the World Health Organization (WHO) cut-off was applied where non-compliance was defined as > 1 h/day and compliance as ≤ 1 h/day [7]. For children aged 6 years and older, since the American Academy of Pediatrics does not establish a strict time limit but advises minimizing recreational screen time and ensuring it does not interfere with sleep, PA, or other essential behaviors [31], a threshold of ≤ 1 h/day was used as an indicator of compliance.

#### Sleep Duration

The sleep duration was evaluated through the questions: “How long does your child sleep at night during weekdays and on weekend days or holidays?” and “How long does your child nap during weekdays and on weekend days or holidays?” Total sleep duration (hours per day) was calculated by summing the hours of nighttime sleep and nap time across weekdays and weekends, and holidays. To obtain a more accurate representation of average sleep duration over the week, a weighted average was applied, with specific weights assigned to each type of day (weekday, weekend, or holiday) based on their frequency in a typical week. Sleep duration (hours/day) and adequacy of the sleep pattern (inadequate; adequate) were obtained. An adequate sleep pattern was considered when it ranged between 10 and 14 h/day for children under 6 years old and between 9 and 12 h/day for children over 6 years old. Sleeping less or more than these values was considered an inadequate sleep pattern [8].

#### Statistical Analyses

Continuous variables were presented as mean ± standard deviation (SD). Data distribution was assessed using the Kolmogórov-Smirnov test. Differences between girls and boys in time spent at sedentary and different PA intensities, physical activities related to an active lifestyle (outdoor playtime preferences, non-continuous walks of at least 30 min daily, outdoor family activities at least once a week, and activity preferences such as running vs. sedentary activities like sitting or playing with toys), screen time, and sleep time were compared using multivariate general linear models adjusting for age, BMI and center. All models were evaluated by model control (normality of residuals and variance homogeneity). For comparison of frequency distributions (lifestyle activities, extracurricular sport activities and screen time evaluated by questionnaires), X2 test was used. This test was conducted stratifying the data according to each extracurricular sport activity.

Given the data dispersion, Spearman's correlation analysis was performed to examine the relationships between age, sedentary time, and time spent at different PA intensities by sex. In contrast, Pearson correlation coefficients were calculated for sleep duration and total screen time in the entire population, as these variables exhibited normal data dispersion. The strength of the correlations was interpreted as follows: weak (rho/r < 0.3), moderate (rho/r = 0.3–0.5), or strong (rho/r > 0.5).

To examine a potential synergy between age and sex in relation to sedentary time and different PA intensity levels, linear regression analyses were performed, incorporating interaction terms. Sedentary time and PA intensity levels were included as dependent variables, with sex, age, and the interaction variable as independent variables. The results were presented using the β standardized coefficients and their statistical significance to assess the strength and relevance of the associations.

Tests were done using a two-sided 5% significance level, and the statistical analyses were conducted using SPSS software version 25 (SPSS Inc., Chicago, IL, USA) and R v.3.6.3 package.

## Results

### Demographic and Anthropometric Data

**Fig. 1:**
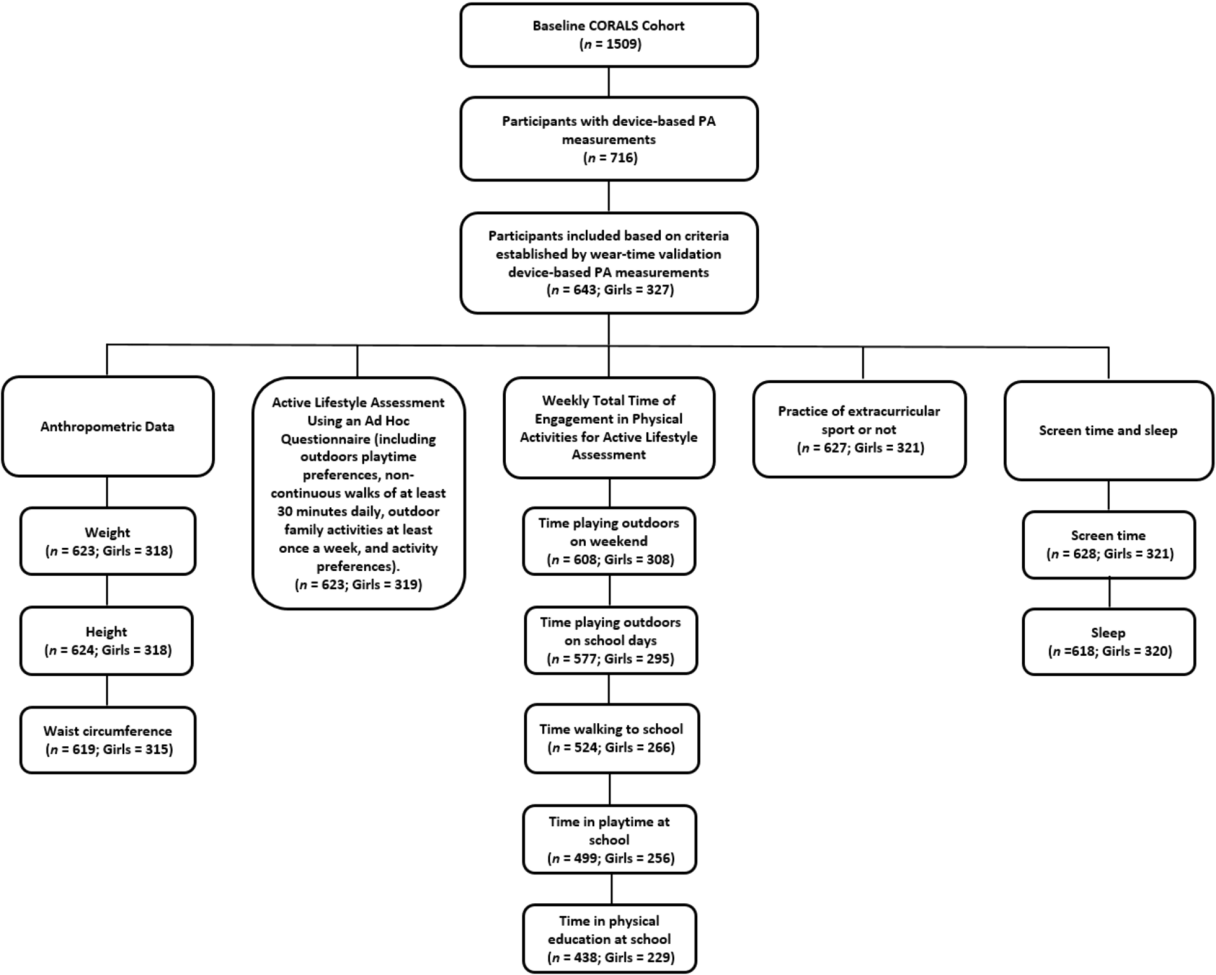
Sample characteristics for each measure and variable, including sex-specific breakdown

From 716 participants that provided device-based data regarding the practice of PA, a total of 643 children (50.8% girls) met the accelerometry inclusion criteria for analysis (Fig. 1). Table 1 shows descriptive characteristics of the total study population and stratified by sex. Girls showed higher fat mass index compared with boys without other differences (Table 1). All details of the sample, including the sample size for girls for each of the measures and variables, are presented in Fig. 1.

**Table 1:** Anthropometric measurements of participants included in this study

Variables	Total	Girls	Boys	*p*-value
Age (years)	5 ± 1	5 ± 1	5 ± 1	0.419
Height (cm)	110.3 ± 8.7	109.8 ± 8.6	110.9 ± 8.8	0.116
Weight (kg)	20.2 ± 4.7	20.2 ± 4.8	20.2 ± 4.6	0.683
BMI Z-Score	0.14 ± 1.27	0.15 ± 1.4	0.13 ± 1.14	0.518
WC (cm)	52.5 ± 7.7	52.9 ± 8.5	52.1 ± 6.8	0.344
Fat mass index	3.9 ± 1.3	4.1 ± 1.4	3.7 ± 1.1	** < 0.001**

Comparisons between boys and girls were adjusted for age, BMI and center. Data are expressed as mean ± standard deviation

*BMI* body mass index, *WC* waist circumference

Values in bold denote statistical significance at *p* 0.05

### Physical Activity Reported by Device-Based Measures

The participants wore the accelerometer for 6.7 ± 0.6 days, with no difference by sex or age. Overall, 67% of preschool children met the general PA recommendations [2, 6, 7], with adherence varying across age groups: 50% in 3-year-olds, 60.5% in 4-year-olds, 74.3% in 5-year-olds, and 73% in 6-year-olds. The participants spent an average of 393.8 ± 65.2 min/day on total PA (girls: 385.8 ± 63.7 min/day *vs* boys: 402 ± 65.9 min/day; *p* = 0.008). No differences in sedentary time were observed by sex, although boys spent more min/day engaged in moderate, vigorous and MVPA compared to girls (Table 2). Preschool children dedicated more time to light and moderate PA and less sedentary time during weekdays than on weekends, with no differences for vigorous PA and MVPA (Table 3).

**Table 2:** Physical activity levels and sedentary time by sex

Variables	Total (n = 643)	Girls (n = 327)	Boys (n = 316)	*p*-value
Sedentary time (min/day)	635.2 ± 124	631.7 ± 124.2	638.8 ± 124	0.419
Light PA (min/day)	321.6 ± 50.1	322.3 ± 50.4	320.9 ± 49.9	0.703
Moderate PA (min/day)	53.5 ± 18.5	47.9 ± 16.4	59.3 ± 18.8	** < 0.001**
Vigorous PA (min/day)	18.6 ± 11.2	15.6 ± 9.2	21.7 ± 12.2	** < 0.001**
MVPA (min/day)	72.1 ± 28.4	63.5 ± 24.3	81 ± 29.5	** < 0.001**

Comparisons between boys and girls were adjusted for age, BMI and center. Data are expressed as mean of min/day ± standard deviation

*MVPA* moderate-vigorous physical activity, *PA* physical activity

Values in bold denote statistical significance at *p* 0.05

**Table 3:** Comparison of physical activity intensities and sedentary time between weekdays and weekends

Variables	Weekdays (n = 643)	Weekend days (n = 643)	*p*-value
Sedentary time (min/day)	625.1 ± 120.1	660.4 ± 164.7	** < 0.001**
Light PA (min/day)	327.7 ± 53.1	306.4 ± 62.8	** < 0.001**
Moderate PA (min/day)	53.9 ± 19.2	52.4 ± 21.8	**0.016**
Vigorous PA (min/day)	18.4 ± 11.5	19.1 ± 14.6	0.850
MVPA (min/day)	72.4 ± 29.1	71.5 ± 34.1	0.198

Comparisons between boys and girls were adjusted for age, BMI and center. Data are expressed as mean of min/day ± standard deviation

*PA* physical activity, *MVPA* moderate-vigorous physical activity

Values in bold denote statistical significance at *p* 0.05

Age displayed a direct correlation with sedentary time (rho = 0.081; *p* = 0.040) and MVPA (rho = 0.183; *p* < 0.001), while showing an inverse correlation with light PA (rho = -0.090; *p* = 0.023). Similarly, age exhibited a direct correlation with sedentary time (rho = 0.086; *p* = 0.030) and MVPA (rho = 0.114; *p* = 0.004) during weekends, and conversely, an inverse relationship with light PA (rho = -0.133; *p* = 0.001). Additionally, on weekdays, there was a direct correlation with age and MVPA (rho = 0.181; *p* < 0.001). Additional details on the correlations are provided (Additional file 1).

**Fig. 2:**
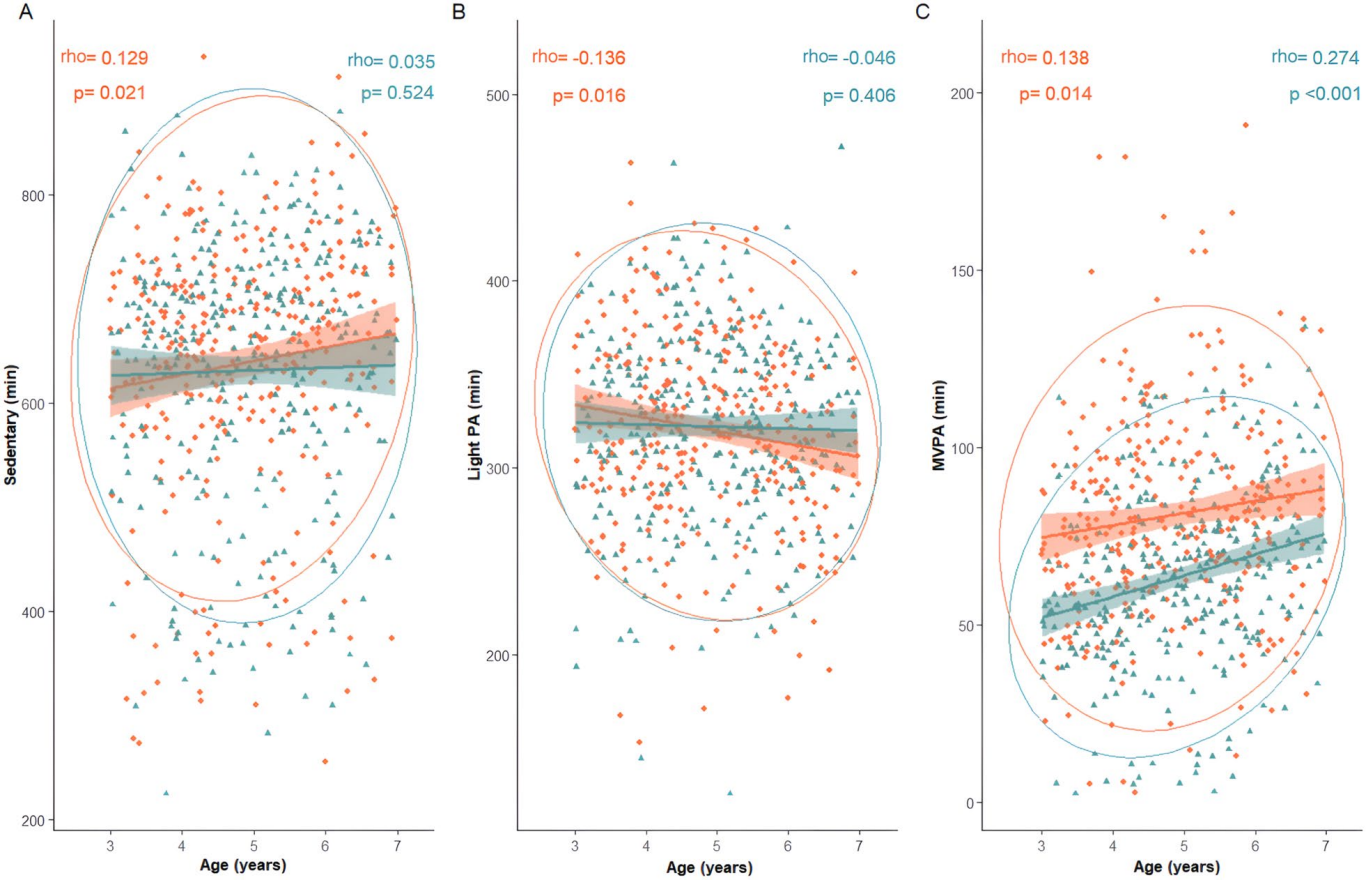
Spearman's correlations between age, sedentary time and time spent at different PA intensities, stratified by sex. (**A** Sedentary; **B** Light PA; **C** MVPA). MVPA: moderate-vigorous physical activity; PA: physical activity. Legend: Each point represents an individual, with girls indicated by green triangles and boys by orange diamonds; the shaded areas illustrate trends for each group; the large circles represent confidence ellipses, showing the distribution and dispersion of the data within each category; the rho and *p* values on each graph indicate the correlation and statistical significance within each group

Regarding sex, older girls exhibited the highest duration of MVPA, with no correlation between sedentary time and light PA with age (Fig. 2). Furthermore, among boys, there was a direct correlation between sedentary time and MVPA with age, while an inverse correlation was observed for light PA (Fig. 2).

Considering age and sex, there was a notable direct correlation between age and MVPA for both boys and girls during weekdays (rho = 0.143;* p* = 0.011; rho = 0.265; *p* < 0.001, respectively), and for girls during weekends (rho = 0.265; *p* < 0.001). However, on weekends, a direct correlation was observed between age and sedentary time in boys (rho = 0.129; *p* = 0.022), while an inverse correlation was noted regarding age and light PA in girls (rho = − 0.131; *p* = 0.020).

According to the regression models, age showed a significant relationship with sedentary time, whereas sex and the age-sex interaction were not significant factors. In the light PA model, sex was significantly associated with the time spent in this activity, but age and the interaction were not significant. For moderate PA, sex had a significant relationship, while age and the interaction remained not significant. In the vigorous and MVPA models, both sex and age showed significant associations with activity time, though their interaction was not relevant (Table 4).

**Table 4:** β standardized coefficients and p-values of regression models for sedentary time and physical activity time at different intensities

Variables	β standardized coefficient	*p*-value
*Model 1. Sedentary time (min/day)*		
Sex	0.180	0.340
Age	0.111	**0.047**
Sex-age interaction	0.221	0.253
*Model 2. Light PA time (min/day)*		
Sex	0.271	**0.013**
Age	0.145	0.083
Sex-age interaction	0.302	**0.008**
*Model 3. Moderate PA time (min/day)*		
Sex	0.573	**0.001**
Age	0.077	0.144
Sex-age interaction	0.273	0.136
*Model 4. Vigorous PA time (min/day)*		
Sex	0.406	**0.022**
Age	0.191	** < 0.001**
Sex-age interaction	0.134	0.462
*Model 5. MVPA time (min/day)*		
Sex	0.535	**0.003**
Age	0.126	**0.016**
Sex-age interaction	0.231	0.203

*PA* physical activity, *MVPA* moderate-vigorous physical activity

Values in bold denote statistical significance at *p* 0.05

### Results Reported by Questionnaires

#### Active Lifestyle

Parent-reported questionnaires showed that children allocated 105 ± 56.2 min/week on motor skills activities and physical education at school, and 162.4 ± 113.9 min/week on playtime at school. Children who engaged in non-continuous walks of at least 30 min daily had lower sedentary time compared to those who did not (632.9 ± 124 min/day *vs.* 667.2 ± 112 min/day; *p* = 0.023). Similarly, lower sedentary time was observed in children who participated in outdoor family activities at least once a week (630.3 ± 123.1 min/day *vs.* 655.2 ± 122.5 min/day;* p* = 0.025). Children who played outdoors at least three days a week had higher levels of light PA compared to those who played outdoors less frequently (323.5 ± 49.4 min/day *vs.* 311.2 ± 52.6 min/day;* p* = 0.015). Similarly, children who engaged in active play, such as running, had higher MVPA time compared to those who preferred sedentary activities like sitting, playing with toys, or coloring (74.1 ± 29.3 min/day *vs.* 66.1 ± 24.7 min/day; *p* = 0.001).

Based on sex comparisons, boys dedicated more time to outdoor PA, both on school days and weekends (Table 5).

**Table 5:** Weekly total time of engagement in physical activities for active lifestyle assessment compared by sex assessment by questionnaires

Variables	Total	Girls	Boys	*p*-value
Walking to school (min/day)	10 ± 10.9	10 ± 8.2	10 ± 13.2	0.709
Playing outdoor on school days (min/day)	98.5 ± 66.6	91.8 ± 64.8	105.6 ± 67.8	**0.003**
Playing outdoor on weekend (min/day)	199.3 ± 120.8	184.5 ± 113.8	214.4 ± 126	**0.009**

Comparisons between boys and girls were adjusted for age, BMI and center. Data are expressed as means of min/day ± standard deviation

Values in bold denote statistical significance at *p* 0.05

#### Practice of Extracurricular Sports

According to the questionnaires, 67.5% of children participated in at least one extracurricular sport activity, with no significant differences between sexes (girls: 65.6% *vs.* boys: 70.7%; *p* = 0.168) or in the time spent (girls: 141.6 ± 90.6 min/week *vs.* boys: 142.3 ± 83.7 min/week; *p* = 0.555), and swimming emerged as the most commonly practiced sport. Girls chose dancing and gymnastics as the most preferred disciplines, whereas boys showed a diverse interest in sports including football, martial arts, ball sports (basketball, volleyball, and handball) and racket sports. A higher duration of MVPA and reduced sedentary time was linked with extracurricular aerobic activities, such as football, basketball, swimming, and athletics, compared to strength and flexibility activities including dance, yoga, or martial arts (74.5 ± 27.7 min/day *vs.* 69.1 ± 29 min/day;* p* = 0.002; 585.1 ± 147.6 min/day *vs.* 534.9 ± 248.6 min/day;* p* = 0.043, respectively) (Additional file 2).

#### Screen Time and Sleep

Based on questionnaire responses related to sedentary behaviors, preschoolers spent an average of 1.8 ± 1 h/day on screen time. 42.1% of preschoolers complied with the recommendations for less than 1 h per day of screen time during weekdays, while only 9.5% met this recommendation on weekends. There were no differences in the time spent on sedentary behavior and MVPA based on whether preschoolers met the screen time recommendations or not (Table 6).

**Table 6:** Percentages of daily screen time stratified by sedentary time, MVPA and sex reported by questionnaires

Variables	Total	Girls	Boys	*p*-value
*Screen time in weekdays (%)*				
Less than 1 h/day	42.1	43.9	40.1	0.296
More than 1 h/day	57.9	56.1	59.9	0.794
*p*-value*	** < 0.001**	**0.029**	**0.001**	
*Screen time in weekends (%)*				
Less than 1 h/day	9.5	11.1	7.8	0.121
More than 1 h/day	90.5	88.9	92.2	0.867
*p*-value*	** < 0.001**	** < 0.001**	** < 0.001**	
*Sedentary and MVPA according to the screen time cut-offs*				
*Sedentary time (min/day)*				
Less than 1 h/day	641.2 ± 120.8	644.6 ± 122.6	637.3 ± 119	0.621
More than 1 h/day	631 ± 126.5	621.3 ± 125.3	640.9 ± 127.2	0.140
*p*-value*	0.317	0.095	0.800	
*MVPA time (min/day)*				
Less than 1 h/day	69.8 ± 29.3	62 ± 25.7	78.7 ± 30.7	** < 0.001**
More than 1 h/day	74.2 ± 27.3	65.4 ± 22.4	82.7 ± 29	** < 0.001**
*p*-value*	0.058	0.213	0.250	

Comparisons between boys and girls were adjusted for age, BMI and center. Data are expressed as percentage or mean of min/day ± standard deviation; *p*-value: comparison between sexes. *p*-value*: comparison between time less or more than 1 h/day

*MVPA* moderate-vigorous physical activity

Values in bold denote statistical significance at *p* 0.05

In the assessment of sleep behaviors, 67.3% of preschoolers met the sleep recommendations for this stage, having an average sleep duration of 10 ± 1 h/day. Only 16.8% reported sleeping < 9 h/day. Furthermore, there were differences in sleep duration between weekdays and weekends (10 ± 0.8 h/day *vs*. 10.2 ± 1.2 h/day; *p* < 0.001, respectively). No differences in sleep duration were observed by sex.

There was a significant inverse correlation between screen time and sleep duration (r = − 0.126, *p* = 0.002), indicating that higher screen time was related to shorter sleep duration.

## Discussion

This study provides device-based insights into the intensity levels and duration of PA and sedentary lifestyle, and an approach to understanding lifestyle behaviors among Spanish preschool-aged children. The main findings reveal that 67% of the preschoolers met the international PA recommendations [2, 6, 7], accumulating an average of 72.1 min/day of MVPA and 393.8 min/day of total PA.

These results are consistent with recent data from a meta-analysis of 48 studies (using device-based measures), including 20,078 preschool-aged children, showing that 60% of children aged 3–5 years meet the international PA recommendations [17]. However, there is considerable variability in the prevalence estimates of preschool-aged children adhering to the international PA guidelines [2, 6, 7], with device-based studies assessing PA reporting different percentages. For example, in European children aged 2–10 years, including Spanish children, the reported percentage achieving 60 min of MVPA was around 20% [32]. In contrast, 84% of Canadian children aged 3–4 years were reported to meet the PA guidelines of 180 min of total PA daily [33], and U.S. preschool-aged children were found to engage in an average of 361 min of PA per day [34]. The discrepancy in these findings may be attributed to the different accelerometry protocols used to assess PA. In fact, studies using device-based measures with preschool-aged children (none of which included Spanish children) consistently report high levels of sedentary time and low levels of PA, with compliance to the recommended PA guidelines often below 20% [35–41]. The lack of international consensus on accelerometer cut-off points for different age ranges during childhood is a significant limitation in PA research, leading to substantial variability in the estimation of prevalence of children adhering to recommendations [17, 42].

Several studies have reported low levels of compliance with guidelines for MVPA [32, 35, 37, 43], although these studies did not include device-based measurements [43]. Furthermore, in the present study, higher light PA practice was observed compared with other studies compiled in a systematic review [44]. Moreover, some authors have noted that collecting data during the winter season may be a limitation to consider [35, 37]. However, in our study, data were primarily collected during the school year, considering time spent both indoors and outdoors. These environmental factors may play a role in the engagement of PA and sedentary behavior patterns [45, 46] and may contribute to the variations in preschoolers' PA levels across different countries [47]. While seasonal variations can influence PA levels in other regions [48], there is a lack of data regarding the variation in PA according to climate in preschool children in Spain. This gap in the literature suggests the need for future research to explore how climate-related factors may affect preschoolers' PA patterns in this specific context.

Sex differences were also observed in both duration and intensity levels of PA. Preschool boys spent more time engaged in moderate, vigorous, and MVPA compared to girls, as has been previously reported in this stage of life [34, 49–54].

In terms of sedentary time and PA time during weekdays and weekends, our sample exhibited greater sedentary time on weekends and higher levels of light and moderate PA during weekdays. These findings align with those reported in other studies involving preschool children [55], probably due to structured PA-related behaviors on weekdays [56].

The questionnaires are a useful complementary tool to evaluate the active lifestyle and to obtain specific information about the diversity of extracurricular sports and activities associated to the time and intensity for PA in these children [12]. In the present study, about 60% of the participants engaged in extracurricular sports activities similar as the results reported in another Spanish study [43]. Boys were more involved than girls in sports that may potentially yield higher levels of MVPA, such as football and ball sports (basketball, volleyball, and handball), as observed in previous research [57, 58]. It has been reported that girls tend to participate less in organized sports, receive less social support for engaging in PA, and perceive less enjoyment in physical education [59]. Moreover, the family's influence on participation in PA may differ based on sex. Boys may receive more support and encouragement to engage in physical activities compared to girls, potentially influencing their activity levels [60]. Moreover, during school breaks or after-school programs, boys tend to use this time to engage in active playtime and sports, while girls often participate in sedentary activities focused on social interactions [43, 61–64].

Older children, even within the preschool age group, exhibit a distinct pattern of PA compared to their younger peers. Girls consistently exhibit higher intensity PA, encompassing both moderate and vigorous activities, while maintaining relatively stable levels of sedentary behavior and light PA. In contrast, boys show no significant age-related changes in moderate PA but tend to spend more time in sedentary activities and vigorous PA. Interestingly, the collective analysis of MVPA reveals a direct correlation with age for both sexes, primarily influenced by an escalation in vigorous PA among boys. These low, but statistically significant correlations, might show a trend of association between these variables, providing insights to guide future research directions within this cohort. Therefore, these nuanced sex-specific differences in PA patterns across age groups highlight the need for targeted interventions aimed to promoting PA in both boys and girls [65]. Consistent with the findings of other researchers, who identify a decline in PA from the age of seven in both sexes [66], further research exploring socio-cultural and environmental factors shaping sex-specific PA trends during early childhood can provide valuable insights. To effectively promote interventions and strategies to increase PA in preschool children, it is important to consider sex differences [49], ensure variability in sports offerings [67], and account for children’s desire for physical challenges [68].

Cultural perspectives play a significant role in shaping PA patterns in preschool children. They influence attitudes, beliefs, and practices surrounding PA, and therefore impact children's engagement [69]. In Spain, the cultural influences described in the literature appear to have a less pronounced impact on preschoolers' PA patterns compared to other countries. Spain's cultural context promotes a balanced approach to child development, with PA and outdoor play widely encouraged for both boys and girls [70]. The Mediterranean lifestyle, coupled with favorable weather conditions, contribute to consistent engagement in physical activities. Additionally, Spanish society tends to minimize gender-based restrictions on PA, promoting an equitable participation [71]. These factors may explain why a high percentage of Spanish preschoolers in our study met international PA recommendations, suggesting that cultural barriers observed in other settings are less influential in Spain.

Regarding sedentary behavior, the WHO [7] propose that in children aged 2 to 5, more than an hour on non-educational activities specially in screen time per day can be considered as excessive. In our study, half of the children met the recommendations, with only 13% adhering to them on weekends. A recent meta-analysis [72] indicated that only one-third of the children aged 2–5 years complies with these recommendations for screen time. Moreover, studies have observed that boys spend more time watching television than girls, both on school days and weekends [73, 74] and are more likely to play physically active games, as well as computer games, compared to girls [75]. As children grow, screen time tends to increase [76, 77], often influenced by parental behavior [78–80].

Data reported from the questionnaires showed that children in the present study slept an average of 10 h per day, in line with the international guidelines (10–13 h/day of sleep [8]. However, it is noteworthy that approximately one-third of the children did not meet these sleep recommendations, as previously reported [81, 82]. Based on the results of this study and previous research [81], increased screen time on portable electronic devices has been linked to reduced daily sleep duration.

The present study provides device-based data on PA and sedentary time, along with a systematic assessment of lifestyle patterns in preschool children in Spain, using a large sample size. Moreover, this cohort study was conducted across different geographical areas in Spain, trying to diversify the sample. However, some limitations must be acknowledged. The cut-off points used, based on those established by Butte et al. [29], for preschoolers, were chosen because their protocol has been specifically validated for this age group, is widely used in the literature, and is included by default in the ActiLife software. Additionally, it is based on the magnitude vector, which provides a more comprehensive assessment of movement intensity. However, these cut-off points could influence the results if compared with those from other accelerometry protocols. Some children did not comply with the accelerometry protocol, being this percentage relatively low (10.2%). Furthermore, the absence of seasonal data and the lack of qualitative insights into children's and families' perceptions and attitudes toward PA may limit a comprehensive understanding of the observed patterns. Additionally, data derived from self-administered questionnaires should be interpreted with caution due to their inherently lower reliability. The use of an ad hoc questionnaire for assessing screen time and sleep patterns could limit comparability with findings from studies employing other available and standardized tools. Moreover, although the study includes data on screen and sleep time, the measurement methods used were not sufficiently rigorous to accurately assess potential associations between screen use and sleep duration. As a result, any conclusions regarding the impact of screen time on sleep should be considered speculative. Future studies with more precise and standardized measurement methods for both screen use and sleep are needed to further explore this relationship. Furthermore, the cross-sectional design limits the ability to examine longitudinal changes in PA patterns as children grow. Future longitudinal studies would be valuable to better understand how PA practice evolves over time in preschool-aged children and explore potential developmental trends across different age groups.

## Conclusions

A significant proportion of Spanish preschoolers meet the international PA recommendations, with variations influenced by sex, age, and days of the week, aligning with global trends at this stage of life. Future efforts should focus on maintaining adequate PA levels and improving adherence. The implementation of sex-specific strategies that consider sport preferences could be particularly beneficial for girls, promoting a more inclusive and effective encouragement of active lifestyles from an early age.

## Supplementary Information

Additional file 1.

Additional file 2.

## Data Availability

The datasets generated and analyzed during the current study are not publicly available due to data regulations and for ethical reasons, considering that this information might compromise research participants’ consent because our participants only gave their consent for the use of their data by the original team of investigators. However, collaboration for data analyses can be requested by sending a letter to the CORALS Steering Committee (estudiocoral@corals.es). The request will then be passed to all the members of the CORALS Steering Committee for deliberation.

## Abbreviations

BMI: Body mass index
CORALS: Childhood Obesity Risk Assessment Longitudinal Study
CPM: Counts per min
ISAK: International Society for the Advancement of Kinanthropometry
MVPA: Moderate-vigorous physical activity
PA: Physical activity
SD: Standard deviation
STROBE: Strengthening the Reporting of Observational Studies in Epidemiology
WC: Waist circumference
WHO: World Health Organization
