# Supplementary material for: Evaluation of Physical Activity, Sedentary Patterns, and Lifestyle Behavior in Spanish Preschool Children from the CORALS Cohort
Source: Sports Med Open. 2025 Jun 9;11:71. doi: 10.1186/s40798-025-00865-2 (PMC12149387; doi:10.1186/s40798-025-00865-2)
Supplement: Supplementary file 1 — Additional file 1. [file 40798_2025_865_MOESM1_ESM.docx]

**Additional File 1.** Spearman's correlations matrix between age, sedentary time and time spent at different PA intensities, stratified by sex.

| **Additional File 1.1.** Spearman's correlations matrix between age, sedentary time and time spent at different PA intensities | | | | | | | | |
| --- | --- | --- | --- | --- | --- | --- | --- | --- |
|  | | | Age of the participant | Average daily time Sedentary lifestyle | Average daily time of light physical activity | Average daily time Moderate Physical Activity | Average daily time Vigorous Physical Activity | Average daily time for moderate and vigorous physical activity |
| Spearman's Rho | Age | Correlation coefficient | 1,000 | ,081 ^*^ | -,090 ^*^ | ,145 ^**^ | ,219 ^**^ | ,183 ^**^ |
|  |  | Sig (bilateral) | . | ,041 | ,023 | ,000 | ,000 | ,000 |
|  |  | N | 643 | 643 | 643 | 643 | 643 | 643 |
|  | Average daily time Sedentary | Correlation coefficient | ,081 ^*^ | 1,000 | -,195 ^**^ | -,229 ^**^ | -,186 ^**^ | -,226 ^**^ |
|  |  | Sig (bilateral) | ,041 | . | ,000 | ,000 | ,000 | ,000 |
|  |  | N | 643 | 643 | 643 | 643 | 643 | 643 |
|  | Average daily time of light physical activity | Correlation coefficient | -,090 ^*^ | -,195 ^**^ | 1,000 | ,405 ^**^ | ,150 ^**^ | ,320 ^**^ |
|  |  | Sig (bilateral) | ,023 | ,000 | . | ,000 | ,000 | ,000 |
|  |  | N | 643 | 643 | 643 | 643 | 643 | 643 |
|  | Average daily time of moderate physical activity | Correlation coefficient | ,145 ^**^ | -,229 ^**^ | ,405 ^**^ | 1,000 | ,817 ^**^ | ,971 ^**^ |
|  |  | Sig (bilateral) | ,000 | ,000 | ,000 | . | ,000 | ,000 |
|  |  | N | 643 | 643 | 643 | 643 | 643 | 643 |
|  | Average daily time of vigorous physical activity | Correlation coefficient | ,219 ^**^ | -,186 ^**^ | ,150 ^**^ | ,817 ^**^ | 1,000 | ,927 ^**^ |
|  |  | Sig (bilateral) | ,000 | ,000 | ,000 | ,000 | . | ,000 |
|  |  | N | 643 | 643 | 643 | 643 | 643 | 643 |
|  | Average daily time of moderate to vigorous physical activity | Correlation coefficient | ,183 ^**^ | -,226 ^**^ | ,320 ^**^ | ,971 ^**^ | ,927 ^**^ | 1,000 |
|  |  | Sig (bilateral) | ,000 | ,000 | ,000 | ,000 | ,000 | . |
|  |  | N | 643 | 643 | 643 | 643 | 643 | 643 |
| *. The correlation is significant at the 0.05 level (two-tailed). | | | | | | | | |
| **. The correlation is significant at the 0.01 level (two-tailed). | | | | | | | | |

| **Additional File 1. 2.** Spearman's correlations matrix between age, sedentary time and time spent at different PA intensities in weekdays. | | | | | | | | |
| --- | --- | --- | --- | --- | --- | --- | --- | --- |
|  | | | Age of the participant | Average daily time Sedentary lifestyle weekdays | Average daily time Light Physical Activity weekdays | Average daily time of moderate physical activity on weekdays | Average daily time Vigorous Physical Activity weekdays | Average daily time for Moderate and Vigorous Physical Activity on weekdays |
| Spearman's Rho | Age of the participant | Correlation coefficient | 1,000 | ,057 | -,068 | ,142 ^**^ | ,211 ^**^ | ,181 ^**^ |
|  |  | Sig (bilateral) | . | ,148 | ,085 | ,000 | ,000 | ,000 |
|  |  | N | 1508 | 643 | 643 | 643 | 643 | 643 |
|  | Average daily time Sedentary lifestyle weekdays | Correlation coefficient | ,057 | 1,000 | -,226 ^**^ | -,249 ^**^ | -,158 ^**^ | -,229 ^**^ |
|  |  | Sig (bilateral) | ,148 | . | ,000 | ,000 | ,000 | ,000 |
|  |  | N | 643 | 643 | 643 | 643 | 643 | 643 |
|  | Average daily time Light Physical Activity weekdays | Correlation coefficient | -,068 | -,226 ^**^ | 1,000 | ,404 ^**^ | ,159 ^**^ | ,325 ^**^ |
|  |  | Sig (bilateral) | ,085 | ,000 | . | ,000 | ,000 | ,000 |
|  |  | N | 643 | 643 | 643 | 643 | 643 | 643 |
|  | Average daily time of moderate physical activity on weekdays | Correlation coefficient | ,142 ^**^ | -,249 ^**^ | ,404 ^**^ | 1,000 | ,823 ^**^ | ,971 ^**^ |
|  |  | Sig (bilateral) | ,000 | ,000 | ,000 | . | ,000 | ,000 |
|  |  | N | 643 | 643 | 643 | 643 | 643 | 643 |
|  | Average daily time Vigorous Physical Activity weekdays | Correlation coefficient | ,211 ^**^ | -,158 ^**^ | ,159 ^**^ | ,823 ^**^ | 1,000 | ,929 ^**^ |
|  |  | Sig (bilateral) | ,000 | ,000 | ,000 | ,000 | . | ,000 |
|  |  | N | 643 | 643 | 643 | 643 | 643 | 643 |
|  | Average daily time for Moderate and Vigorous Physical Activity on weekdays | Correlation coefficient | ,181 ^**^ | -,229 ^**^ | ,325 ^**^ | ,971 ^**^ | ,929 ^**^ | 1,000 |
|  |  | Sig (bilateral) | ,000 | ,000 | ,000 | ,000 | ,000 | . |
|  |  | N | 643 | 643 | 643 | 643 | 643 | 643 |
| **. The correlation is significant at the 0.01 level (two-tailed). | | | | | | | | |

| **Additional File 1. 3.** Spearman's correlations matrix between age, sedentary time and time spent at different PA intensities on weekends. | | | | | | | | |  |
| --- | --- | --- | --- | --- | --- | --- | --- | --- | --- |
|  | | | Age of the participant | Average daily time Sedentary lifestyle weekend days | Average daily time Light Physical Activity weekend days | Average daily time of moderate physical activity on weekend days | Average daily time Vigorous Physical Activity weekend days | Average daily time for Moderate and Vigorous Physical Activity on weekend days | |
| Spearman's Rho | Age of the participant | Correlation coefficient | 1,000 | ,086 ^*^ | -,133 ^**^ | ,100 ^*^ | ,127 ^**^ | ,114 ^**^ | |
|  |  | Sig (bilateral) | . | ,030 | ,001 | ,011 | ,001 | ,004 | |
|  |  | N | 1508 | 643 | 643 | 643 | 643 | 643 | |
|  | Average daily time Sedentary lifestyle weekend days | Correlation coefficient | ,086 ^*^ | 1,000 | -,148 ^**^ | -,189 ^**^ | -,209 ^**^ | -,214 ^**^ | |
|  |  | Sig (bilateral) | ,030 | . | ,000 | ,000 | ,000 | ,000 | |
|  |  | N | 643 | 643 | 643 | 643 | 643 | 643 | |
|  | Average daily time Light Physical Activity weekend days | Correlation coefficient | -,133 ^**^ | -,148 ^**^ | 1,000 | ,464 ^**^ | ,168 ^**^ | ,363 ^**^ | |
|  |  | Sig (bilateral) | ,001 | ,000 | . | ,000 | ,000 | ,000 | |
|  |  | N | 643 | 643 | 643 | 643 | 643 | 643 | |
|  | Average daily time of moderate physical activity on weekend days | Correlation coefficient | ,100 ^*^ | -,189 ^**^ | ,464 ^**^ | 1,000 | ,768 ^**^ | ,958 ^**^ | |
|  |  | Sig (bilateral) | ,011 | ,000 | ,000 | . | ,000 | ,000 | |
|  |  | N | 643 | 643 | 643 | 643 | 643 | 643 | |
|  | Average daily time Vigorous Physical Activity weekend days | Correlation coefficient | ,127 ^**^ | -,209 ^**^ | ,168 ^**^ | ,768 ^**^ | 1,000 | ,909 ^**^ | |
|  |  | Sig (bilateral) | ,001 | ,000 | ,000 | ,000 | . | ,000 | |
|  |  | N | 643 | 643 | 643 | 643 | 643 | 643 | |
|  | Average daily time for Moderate and Vigorous Physical Activity on weekend days | Correlation coefficient | ,114 ^**^ | -,214 ^**^ | ,363 ^**^ | ,958 ^**^ | ,909 ^**^ | 1,000 | |
|  |  | Sig (bilateral) | ,004 | ,000 | ,000 | ,000 | ,000 | . | |
|  |  | N | 643 | 643 | 643 | 643 | 643 | 643 | |
| *. The correlation is significant at the 0.05 level (two-tailed). | | | | | | | | |  |
| **. The correlation is significant at the 0.01 level (two-tailed). | | | | | | | | |  |

| **Additional File 1.4.** Spearman's correlations matrix between age, sedentary time and time spent at different PA intensities, stratified by sex. | | | | | | | | | | | | | | | |  |
| --- | --- | --- | --- | --- | --- | --- | --- | --- | --- | --- | --- | --- | --- | --- | --- | --- |
|  | Sex | | | Age of the participant | | Average daily time Sedentary lifestyle | | Average daily time of light physical activity | | Average daily time Moderate Physical Activity | | Average daily time Vigorous Physical Activity | | Average daily time for moderate and vigorous physical activity | |  |
| Spearman's Rho | Boys | Age of the participant | Correlation coefficient | | 1,000 | | ,129 ^*^ | | -,136 ^*^ | | ,101 | | ,181 ^**^ | | ,138 ^*^ | |
|  |  |  | Sig (bilateral) | | . | | ,021 | | ,016 | | ,073 | | ,001 | | ,014 | |
|  |  |  | N | | 316 | | 316 | | 316 | | 316 | | 316 | | 316 | |
|  |  | Average daily time Sedentary lifestyle | Correlation coefficient | | ,129 ^*^ | | 1,000 | | -,189 ^**^ | | -,305 ^**^ | | -,273 ^**^ | | -,309 ^**^ | |
|  |  |  | Sig (bilateral) | | ,021 | | . | | ,001 | | ,000 | | ,000 | | ,000 | |
|  |  |  | N | | 316 | | 316 | | 316 | | 316 | | 316 | | 316 | |
|  |  | Average daily time of light physical activity | Correlation coefficient | | -,136 ^*^ | | -,189 ^**^ | | 1,000 | | ,409 ^**^ | | ,184 ^**^ | | ,337 ^**^ | |
|  |  |  | Sig (bilateral) | | ,016 | | ,001 | | . | | ,000 | | ,001 | | ,000 | |
|  |  |  | N | | 316 | | 316 | | 316 | | 316 | | 316 | | 316 | |
|  |  | Average daily time Moderate Physical Activity | Correlation coefficient | | ,101 | | -,305 ^**^ | | ,409 ^**^ | | 1,000 | | ,810 ^**^ | | ,967 ^**^ | |
|  |  |  | Sig (bilateral) | | ,073 | | ,000 | | ,000 | | . | | ,000 | | ,000 | |
|  |  |  | N | | 316 | | 316 | | 316 | | 316 | | 316 | | 316 | |
|  |  | Average daily time Vigorous Physical Activity | Correlation coefficient | | ,181 ^**^ | | -,273 ^**^ | | ,184 ^**^ | | ,810 ^**^ | | 1,000 | | ,927 ^**^ | |
|  |  |  | Sig (bilateral) | | ,001 | | ,000 | | ,001 | | ,000 | | . | | ,000 | |
|  |  |  | N | | 316 | | 316 | | 316 | | 316 | | 316 | | 316 | |
|  |  | Average daily time for moderate and vigorous physical activity | Correlation coefficient | | ,138 ^*^ | | -,309 ^**^ | | ,337 ^**^ | | ,967 ^**^ | | ,927 ^**^ | | 1,000 | |
|  |  |  | Sig (bilateral) | | ,014 | | ,000 | | ,000 | | ,000 | | ,000 | | . | |
|  |  |  | N | | 316 | | 316 | | 316 | | 316 | | 316 | | 316 | |
|  | Girls | Age of the participant | Correlation coefficient | | 1,000 | | ,035 | | -,046 | | ,231 ^**^ | | ,298 ^**^ | | ,274 ^**^ | |
|  |  |  | Sig (bilateral) | | . | | ,524 | | ,406 | | ,000 | | ,000 | | ,000 | |
|  |  |  | N | | 327 | | 327 | | 327 | | 327 | | 327 | | 327 | |
|  |  | Average daily time Sedentary lifestyle | Correlation coefficient | | ,035 | | 1,000 | | -,197 ^**^ | | -,198 ^**^ | | -,136 ^*^ | | -,187 ^**^ | |
|  |  |  | Sig (bilateral) | | ,524 | | . | | ,000 | | ,000 | | ,014 | | ,001 | |
|  |  |  | N | | 327 | | 327 | | 327 | | 327 | | 327 | | 327 | |
|  |  | Average daily time of light physical activity | Correlation coefficient | | -,046 | | -,197 ^**^ | | 1,000 | | ,478 ^**^ | | ,172 ^**^ | | ,377 ^**^ | |
|  |  |  | Sig (bilateral) | | ,406 | | ,000 | | . | | ,000 | | ,002 | | ,000 | |
|  |  |  | N | | 327 | | 327 | | 327 | | 327 | | 327 | | 327 | |
|  |  | Average daily time Moderate Physical Activity | Correlation coefficient | | ,231 ^**^ | | -,198 ^**^ | | ,478 ^**^ | | 1,000 | | ,791 ^**^ | | ,966 ^**^ | |
|  |  |  | Sig (bilateral) | | ,000 | | ,000 | | ,000 | | . | | ,000 | | ,000 | |
|  |  |  | N | | 327 | | 327 | | 327 | | 327 | | 327 | | 327 | |
|  |  | Average daily time Vigorous Physical Activity | Correlation coefficient | | ,298 ^**^ | | -,136 ^*^ | | ,172 ^**^ | | ,791 ^**^ | | 1,000 | | ,915 ^**^ | |
|  |  |  | Sig (bilateral) | | ,000 | | ,014 | | ,002 | | ,000 | | . | | ,000 | |
|  |  |  | N | | 327 | | 327 | | 327 | | 327 | | 327 | | 327 | |
|  |  | Average daily time for moderate and vigorous physical activity | Correlation coefficient | | ,274 ^**^ | | -,187 ^**^ | | ,377 ^**^ | | ,966 ^**^ | | ,915 ^**^ | | 1,000 | |
|  |  |  | Sig (bilateral) | | ,000 | | ,001 | | ,000 | | ,000 | | ,000 | | . | |
|  |  |  | N | | 327 | | 327 | | 327 | | 327 | | 327 | | 327 | |
| *. The correlation is significant at the 0.05 level (two-tailed). | | | | | | | | | | | | | | | |  |
| **. The correlation is significant at the 0.01 level (two-tailed). | | | | | | | | | | | | | | | |  |

| **Additional File 1. 5.** Spearman's correlations matrix between age, sedentary time and time spent at different PA intensities, stratified by sex in weekdays. | | | | | | | | | | | | | | | | |  |
| --- | --- | --- | --- | --- | --- | --- | --- | --- | --- | --- | --- | --- | --- | --- | --- | --- | --- |
| 1 | Sex | | | | Age of the participant | | Average daily time Sedentary lifestyle weekdays | | Average daily time Light Physical Activity weekdays | | Average daily time of moderate physical activity on weekdays | | Average daily time Vigorous Physical Activity weekdays | | Average daily time for Moderate and Vigorous Physical Activity on weekdays | |  |
| Spearman's Rho | Boys | Age of the participant | Correlation coefficient | 1,000 | | ,098 | | -,113 ^*^ | | ,094 | | ,191 ^**^ | | ,143 ^*^ | |  |  |
|  |  |  | Sig (bilateral) | . | | ,082 | | ,044 | | ,095 | | ,001 | | ,011 | |  |  |
|  |  |  | N | 316 | | 316 | | 316 | | 316 | | 316 | | 316 | |  |  |
|  |  | Average daily time Sedentary lifestyle weekdays | Correlation coefficient | ,098 | | 1,000 | | -,201 ^**^ | | -,320 ^**^ | | -,251 ^**^ | | -,315 ^**^ | |  |  |
|  |  |  | Sig (bilateral) | ,082 | | . | | ,000 | | ,000 | | ,000 | | ,000 | |  |  |
|  |  |  | N | 316 | | 316 | | 316 | | 316 | | 316 | | 316 | |  |  |
|  |  | Average daily time Light Physical Activity weekdays | Correlation coefficient | -,113 ^*^ | | -,201 ^**^ | | 1,000 | | ,411 ^**^ | | ,188 ^**^ | | ,344 ^**^ | |  |  |
|  |  |  | Sig (bilateral) | ,044 | | ,000 | | . | | ,000 | | ,001 | | ,000 | |  |  |
|  |  |  | N | 316 | | 316 | | 316 | | 316 | | 316 | | 316 | |  |  |
|  |  | Average daily time of moderate physical activity on weekdays | Correlation coefficient | ,094 | | -,320 ^**^ | | ,411 ^**^ | | 1,000 | | ,797 ^**^ | | ,963 ^**^ | |  |  |
|  |  |  | Sig (bilateral) | ,095 | | ,000 | | ,000 | | . | | ,000 | | ,000 | |  |  |
|  |  |  | N | 316 | | 316 | | 316 | | 316 | | 316 | | 316 | |  |  |
|  |  | Average daily time Vigorous Physical Activity weekdays | Correlation coefficient | ,191 ^**^ | | -,251 ^**^ | | ,188 ^**^ | | ,797 ^**^ | | 1,000 | | ,924 ^**^ | |  |  |
|  |  |  | Sig (bilateral) | ,001 | | ,000 | | ,001 | | ,000 | | . | | ,000 | |  |  |
|  |  |  | N | 316 | | 316 | | 316 | | 316 | | 316 | | 316 | |  |  |
|  |  | Average daily time for Moderate and Vigorous Physical Activity on weekdays | Correlation coefficient | ,143 ^*^ | | -,315 ^**^ | | ,344 ^**^ | | ,963 ^**^ | | ,924 ^**^ | | 1,000 | |  |  |
|  |  |  | Sig (bilateral) | ,011 | | ,000 | | ,000 | | ,000 | | ,000 | | . | |  |  |
|  |  |  | N | 316 | | 316 | | 316 | | 316 | | 316 | | 316 | |  |  |
|  | Girls | Age of the participant | Correlation coefficient | 1,000 | | ,020 | | -,029 | | ,230 ^**^ | | ,272 ^**^ | | ,265 ^**^ | |  |  |
|  |  |  | Sig (bilateral) | . | | ,719 | | ,596 | | ,000 | | ,000 | | ,000 | |  |  |
|  |  |  | N | 327 | | 327 | | 327 | | 327 | | 327 | | 327 | |  |  |
|  |  | Average daily time Sedentary lifestyle weekdays | Correlation coefficient | ,020 | | 1,000 | | -,249 ^**^ | | -,203 ^**^ | | -,088 | | -,169 ^**^ | |  |  |
|  |  |  | Sig (bilateral) | ,719 | | . | | ,000 | | ,000 | | ,112 | | ,002 | |  |  |
|  |  |  | N | 327 | | 327 | | 327 | | 327 | | 327 | | 327 | |  |  |
|  |  | Average daily time Light Physical Activity weekdays | Correlation coefficient | -,029 | | -,249 ^**^ | | 1,000 | | ,473 ^**^ | | ,190 ^**^ | | ,383 ^**^ | |  |  |
|  |  |  | Sig (bilateral) | ,596 | | ,000 | | . | | ,000 | | ,001 | | ,000 | |  |  |
|  |  |  | N | 327 | | 327 | | 327 | | 327 | | 327 | | 327 | |  |  |
|  |  | Average daily time of moderate physical activity on weekdays | Correlation coefficient | ,230 ^**^ | | -,203 ^**^ | | ,473 ^**^ | | 1,000 | | ,814 ^**^ | | ,972 ^**^ | |  |  |
|  |  |  | Sig (bilateral) | ,000 | | ,000 | | ,000 | | . | | ,000 | | ,000 | |  |  |
|  |  |  | N | 327 | | 327 | | 327 | | 327 | | 327 | | 327 | |  |  |
|  |  | Average daily time Vigorous Physical Activity weekdays | Correlation coefficient | ,272 ^**^ | | -,088 | | ,190 ^**^ | | ,814 ^**^ | | 1,000 | | ,920 ^**^ | |  |  |
|  |  |  | Sig (bilateral) | ,000 | | ,112 | | ,001 | | ,000 | | . | | ,000 | |  |  |
|  |  |  | N | 327 | | 327 | | 327 | | 327 | | 327 | | 327 | |  |  |
|  |  | Average daily time for Moderate and Vigorous Physical Activity on weekdays | Correlation coefficient | ,265 ^**^ | | -,169 ^**^ | | ,383 ^**^ | | ,972 ^**^ | | ,920 ^**^ | | 1,000 | |  |  |
|  |  |  | Sig (bilateral) | ,000 | | ,002 | | ,000 | | ,000 | | ,000 | | . | |  |  |
|  |  |  | N | 327 | | 327 | | 327 | | 327 | | 327 | | 327 | |  |  |
| *. The correlation is significant at the 0.05 level (two-tailed). | | | | | | | | | | | | | | | | | |
| **. The correlation is significant at the 0.01 level (two-tailed). | | | | | | | | | | | | | | | | | |

| **Additional File 1. 6** Spearman's correlations matrix between age, sedentary time and time spent at different PA intensities, stratified by sex in weekends. | | | | | | | | | | | | | | | | | |
| --- | --- | --- | --- | --- | --- | --- | --- | --- | --- | --- | --- | --- | --- | --- | --- | --- | --- |
|  | Sex | | | | Age of the participant | | Average daily time Sedentary lifestyle weekend days | | Average daily time Light Physical Activity weekend days | | Average daily time of moderate physical activity on weekend days | | Average daily time Vigorous Physical Activity weekend days | | Average daily time for Moderate and Vigorous Physical Activity on weekend days | | |
| Spearman's Rho | Boys | Age of the participant | Correlation coefficient | 1,000 | | ,128 ^*^ | | -,171 ^**^ | | ,077 | | ,083 | | ,081 | |  |  |
|  |  |  | Sig (bilateral) | . | | ,022 | | ,002 | | ,173 | | ,141 | | ,151 | |  |  |
|  |  |  | N | 742 | | 316 | | 316 | | 316 | | 316 | | 316 | |  |  |
|  |  | Average daily time Sedentary lifestyle weekend days | Correlation coefficient | ,128 ^*^ | | 1,000 | | -,209 ^**^ | | -,266 ^**^ | | -,241 ^**^ | | -,275 ^**^ | |  |  |
|  |  |  | Sig (bilateral) | ,022 | | . | | ,000 | | ,000 | | ,000 | | ,000 | |  |  |
|  |  |  | N | 316 | | 316 | | 316 | | 316 | | 316 | | 316 | |  |  |
|  |  | Average daily time Light Physical Activity weekend days | Correlation coefficient | -,171 ^**^ | | -,209 ^**^ | | 1,000 | | ,446 ^**^ | | ,178 ^**^ | | ,354 ^**^ | |  |  |
|  |  |  | Sig (bilateral) | ,002 | | ,000 | | . | | ,000 | | ,001 | | ,000 | |  |  |
|  |  |  | N | 316 | | 316 | | 316 | | 316 | | 316 | | 316 | |  |  |
|  |  | Average daily time of moderate physical activity on weekend days | Correlation coefficient | ,077 | | -,266 ^**^ | | ,446 ^**^ | | 1,000 | | ,784 ^**^ | | ,962 ^**^ | |  |  |
|  |  |  | Sig (bilateral) | ,173 | | ,000 | | ,000 | | . | | ,000 | | ,000 | |  |  |
|  |  |  | N | 316 | | 316 | | 316 | | 316 | | 316 | | 316 | |  |  |
|  |  | Average daily time Vigorous Physical Activity weekend days | Correlation coefficient | ,083 | | -,241 ^**^ | | ,178 ^**^ | | ,784 ^**^ | | 1,000 | | ,913 ^**^ | |  |  |
|  |  |  | Sig (bilateral) | ,141 | | ,000 | | ,001 | | ,000 | | . | | ,000 | |  |  |
|  |  |  | N | 316 | | 316 | | 316 | | 316 | | 316 | | 316 | |  |  |
|  |  | Average daily time for Moderate and Vigorous Physical Activity on weekend days | Correlation coefficient | ,081 | | -,275 ^**^ | | ,354 ^**^ | | ,962 ^**^ | | ,913 ^**^ | | 1,000 | |  |  |
|  |  |  | Sig (bilateral) | ,151 | | ,000 | | ,000 | | ,000 | | ,000 | | . | |  |  |
|  |  |  | N | 316 | | 316 | | 316 | | 316 | | 316 | | 316 | |  |  |
|  | Girls | Age of the participant | Correlation coefficient | 1,000 | | ,044 | | -,094 | | ,143 ^**^ | | ,189 ^**^ | | ,172 ^**^ | |  |  |
|  |  |  | Sig (bilateral) | . | | ,424 | | ,090 | | ,009 | | ,001 | | ,002 | |  |  |
|  |  |  | N | 766 | | 327 | | 327 | | 327 | | 327 | | 327 | |  |  |
|  |  | Average daily time Sedentary lifestyle weekend days | Correlation coefficient | ,044 | | 1,000 | | -,089 | | -,161 ^**^ | | -,224 ^**^ | | -,206 ^**^ | |  |  |
|  |  |  | Sig (bilateral) | ,424 | | . | | ,109 | | ,003 | | ,000 | | ,000 | |  |  |
|  |  |  | N | 327 | | 327 | | 327 | | 327 | | 327 | | 327 | |  |  |
|  |  | Average daily time Light Physical Activity weekend days | Correlation coefficient | -,094 | | -,089 | | 1,000 | | ,510 ^**^ | | ,177 ^**^ | | ,397 ^**^ | |  |  |
|  |  |  | Sig (bilateral) | ,090 | | ,109 | | . | | ,000 | | ,001 | | ,000 | |  |  |
|  |  |  | N | 327 | | 327 | | 327 | | 327 | | 327 | | 327 | |  |  |
|  |  | Average daily time of moderate physical activity on weekend days | Correlation coefficient | ,143 ^**^ | | -,161 ^**^ | | ,510 ^**^ | | 1,000 | | ,742 ^**^ | | ,949 ^**^ | |  |  |
|  |  |  | Sig (bilateral) | ,009 | | ,003 | | ,000 | | . | | ,000 | | ,000 | |  |  |
|  |  |  | N | 327 | | 327 | | 327 | | 327 | | 327 | | 327 | |  |  |
|  |  | Average daily time Vigorous Physical Activity weekend days | Correlation coefficient | ,189 ^**^ | | -,224 ^**^ | | ,177 ^**^ | | ,742 ^**^ | | 1,000 | | ,901 ^**^ | |  |  |
|  |  |  | Sig (bilateral) | ,001 | | ,000 | | ,001 | | ,000 | | . | | ,000 | |  |  |
|  |  |  | N | 327 | | 327 | | 327 | | 327 | | 327 | | 327 | |  |  |
|  |  | Average daily time for Moderate and Vigorous Physical Activity on weekend days | Correlation coefficient | ,172 ^**^ | | -,206 ^**^ | | ,397 ^**^ | | ,949 ^**^ | | ,901 ^**^ | | 1,000 | |  |  |
|  |  |  | Sig (bilateral) | ,002 | | ,000 | | ,000 | | ,000 | | ,000 | | . | |  |  |
|  |  |  | N | 327 | | 327 | | 327 | | 327 | | 327 | | 327 | |  |  |
| *. The correlation is significant at the 0.05 level (two-tailed). | | | | | | | | | | | | | | | | |  |
| **. The correlation is significant at the 0.01 level (two-tailed). | | | | | | | | | | | | | | | | |  |
