# Supplementary material for: Evaluation of Physical Activity, Sedentary Patterns, and Lifestyle Behavior in Spanish Preschool Children from the CORALS Cohort
Source: Sports Med Open. 2025 Jun 9;11:71. doi: 10.1186/s40798-025-00865-2 (PMC12149387; doi:10.1186/s40798-025-00865-2)
Supplement: Supplementary file 2 — Additional file 2. [file 40798_2025_865_MOESM2_ESM.docx]

| **Additional File 2.** Percentages of boys and girls that practice extracurricular sport activities reported by questionnaires. | | | | |
| --- | --- | --- | --- | --- |
| **Extracurricular sport**  **Activities (%)** | **Total**  **(n= 434)** | **Girls**  **(n= 214)** | **Boys**  **(n= 220)** | ***p*-value** |
| Swimming | 42.2 | 41.6 | 42.7 | .620 |
| Dance | 24.5 | 41.6 | 7.3 | **<.001** |
| Football | 16.9 | 4.2 | 29.5 | **<.001** |
| Gimnastic | 11.2 | 18.7 | 3.6 | **<.001** |
| Martial Arts | 7.6 | 3.3 | 11.8 | **.003** |
| Ball sports (basketball, volleyball, handball) | 6.9 | 3.7 | 10 | **.028** |
| Racket sports | 5.5 | 2.8 | 8.2 | **.029** |
| Endurance sports (triathlon, cycling) | 3.7 | 2.8 | 4.5 | .450 |
| Athletics | 3.7 | 5.6 | 1.8 | **.021** |
| Other sports | 20.8 | 21 | 20.5 | .930 |
| Note: Differences analyzed using *X*^2^ stratified by each sport activity. Data for girls and boys are presented as proportions (%) for each activity stratified by sex. A child may participate in more than one sport. | | | | |
